# Cardiovascular risk management: the success of target level achievement in high- and very high-risk patients in Hungary

**DOI:** 10.1186/s12875-022-01922-5

**Published:** 2022-11-29

**Authors:** Zoltán Jancsó, Orsolya Csenteri, Gergő József Szőllősi, Péter Vajer, Péter Andréka

**Affiliations:** Gottsegen National Cardiovascular Center, Haller u. 29, Budapest, 1096 Hungary

**Keywords:** Cardiovascular risk, Risk management, Target level achievement

## Abstract

**Background:**

The management of risk factors in patients with high cardiovascular risk and its effectiveness is of paramount importance. Over the last decade, several studies have examined the achievement of cardiovascular risk factors’ target levels in Europe. In the present Hungarian study, we assessed the cardiovascular risk level of participants aged 40–65 years and the success of achieving risk factors’ target levels in high- and very high-risk patients. We compared these results with the results of two similar European studies.

**Methods:**

We conducted a cross-sectional study involving 37,778 patients aged 40–65 years from Hungary between 2019 and 2020. Cardiovascular risk levels and target values were set according to the 2016 European Guideline. Target achievement was evaluated for body mass index, waist circumference, blood pressure, total, LDL, and HDL cholesterol, triglyceride, and HbA1c (in diabetics).

**Results:**

For 37,298 patients, all the data were available to determine their cardiovascular risk category. Of these, 23.1% had high and 31.4% had very high cardiovascular risk (men: 27.1 and 39.6%, women: 20.5 and 26.1%, respectively). Achievement of the LDL-C target of 1.8 mmol/l was only 8.0% among very high-risk patients, which was significantly lower than the European average (29%). Achievement of target blood pressure among high-risk patients was better than the European average (63.4% vs. 44.7%, respectively); however, achievement was slightly lower among very high-risk patients compared with the European average (49.4% vs. 58%, respectively). The proportion of patients with type 2 diabetes who achieved a HbA1c below 7% was 57.3% in the high-risk population and 53% in the very high-risk population, which was in line with the European average success rates (58.5 and 54%, respectively). Waist circumference (< 88 cm for women and < 102 cm for men) was achieved by 29.4% of patients in the very high-risk group in our survey, which was lower than the European average of 41%.

**Conclusions:**

The success rate of cardiovascular risk management in Hungary is lower than the European average in several parameters. Furthermore, our data highlight the poor effectiveness of obesity management in Hungary. General practice partnerships may be important sites for positive change.

## Introduction

Cardiovascular diseases are the leading cause of adult morbidity and mortality in developed countries; in Hungary, these rates are higher than the European Union average. Hungary is one of the countries with high cardiovascular risk in Europe [1–3].

Recognition, risk assessment, and treatment of cardiovascular risk factors to target levels play a crucial role in the prevention of cardiovascular diseases [4]. Data from previous Hungarian studies on smaller populations suggest that target level achievement is not adequate in cardiovascular prevention [5, 6]. This is also a problem at the European level. Analysis of several relevant European studies concluded that daily clinical practice in cardiovascular risk factor management is far from optimal as set out by international recommendations. The effectiveness of cardiovascular prevention depends largely on both the relevant drug therapy and the success of lifestyle modifications, which requires a multidisciplinary approach [4, 7].

Due to the nature of the service (primary, continuous, easily accessible), primary care is a key site for cardiovascular prevention. In Hungary, the backbone of primary health care is the general practitioner (GP) system. Until 2021, primary care was provided by isolated general practices consisting of one doctor and one or two practice nurse(s). A significant part of the day-to-day work was the care of acute and chronic patients, with insufficient focus on preventive tasks due to a lack of time and well-defined tasks.

Accordingly, several central initiatives have been established in Hungary over the past 20 years to reduce cardiovascular morbidity and mortality. The most recent and one of the most comprehensive of these initiatives is the ‘Three Generations for Health Programme’, which was launched in 2019 as a government initiative [8]. The programme focuses on providing preventive and continuous care services designed to meet local needs and the mandatory establishment of general practice partnerships. The primary aims of the programme are to assess the risk factors and risk levels for cardiovascular diseases and to launch personalised interventions in a large population, involving GPs and three generations of patients (0–18 years, 40–65 years, and 65+ years).

A total of 143 consortia of 806 general practices in Hungary are participating in the implementation of the programme, which is managed and monitored by the Gottsegen György National Cardiovascular Center (GOKVI), which also provides the information technology (IT) background. The data generated during the implementation of the programme are processed using online IT solutions, which allows for their central evaluation, analysis, and the achievement of research objectives.

The results of this programme have also contributed to the legislative support of the establishment of general practice partnerships in Hungary since 2021. Furthermore, the cardiovascular prevention programme has been fully integrated into the tasks performed by GPs.

The focus of the present study is on the population aged 40–65 years, as this is the age group in which the assessment of cardiovascular risk factors and the determination of risk levels and their management can be conducted in clinical practice according to scientific guidelines developed and applied at a Europe-wide level [1, 9]. The efficient functioning of primary care practices has a key role in achieving these goals.

## Study objectives

In the target population of 40–65-year-old patients included in the programme, individual risk factors determining cardiovascular risk (lifestyle risk factors, body mass index [BMI], waist circumference, CH metabolism, lipid parameters, blood pressure) and any existing medical conditions or family history (family or individual medical history) influencing cardiovascular risk were investigated. Based on these data, we determined the cardiovascular risk level of each patient according to the 2016 European Guidelines on cardiovascular disease prevention in clinical practice. Then, we determined the distribution of risk levels in the study population [1].

The primary objective of the present study was to assess the success of achieving the target levels set by the European Guideline for certain risk parameters (blood pressure, lipid parameters, HbA1c, BMI, waist circumference) among people at high and very high cardiovascular risk, which is a good indicator of cardiovascular risk management in primary and specialized care [1]. Furthermore, we compared the success of target level achievement in Hungary with data from international surveys to put risk management practices in Hungary into a global context.

## Methods

The study sample consisted of patients aged 40–65 years who were electronically reported by general practices to the Three Generations for Health programme. Recruitment of patients aged 40–65 years who agreed to participate in the programme was performed consecutively in general practices during in-person appointments, by telecommunication, or during case management for administrative reasons. The anamnestic data of the participating patients, vital parameters obtained during physical examination performed at the general practices, and the results of relevant laboratory tests were recorded. In all cases, the laboratory measurements were performed in accredited laboratories of the hospital or medical university that served the general practice in daily clinical care.

The data were provided by the participating practices through a dedicated online interface (Icardio) from which data were retrieved in a non-personally identifiable way for analysis. Each data set remained linked to the patient who was recruited, and their identity could be deconstructed by their GP if necessary.

The cardiovascular risk level and the target values were defined according to the 2016 European Guidelines on cardiovascular disease prevention in clinical practice using individual data and family history (e.g., age, sex, smoking habits, atherosclerotic disease, chronic kidney disease, diabetes mellitus, premature cardiovascular disease, familial hypercholesterolaemia), taking into account certain vital parameters (blood pressure, BMI, abdominal circumference) and certain laboratory data (total cholesterol, LDL-cholesterol, HDL-cholesterol, triglycerides, HbA1c) [1]. The risk assessment methodology and the target values are presented in Tables 1 and 2. For some of the target values, minor modifications to the relevant Hungarian cardiovascular prevention guidelines have been made and are indicated in Table 2; where relevant, a risk factor was examined according to both target values [9].

**Table 1:** Cardiovascular risk categories [1].

Categories of cardiovascular risk	Determining factors
Very high risk	Subjects with any of the following: • Documented CVD, clinical or unequivocal on imaging. Documented clinical CVD includes previous AMI, ACS, coronary revascularisation or other arterial revascularisation procedures, stroke, TIA, aortic aneurysm, and PAD. Unequivocally documented CVD on imaging includes plaque(s) on coronary angiography or carotid ultrasound. • DM with target organ damage such as proteinuria or with a major risk factor such as smoking or marked hypercholesterolaemia or marked hypertension. • Severe CKD (GFR < 30 ml/min/1.73 m2). • A calculated SCORE ≥ 10%.
High risk	Subjects with: • Markedly elevated single risk factors, especially cholesterol > 8 mmol/l (e.g., in familial hypercholesterolaemia) or BP ≥180/110 mmHg. • Most other people with DM (except for young people with type 1 DM and without major risk factors who may be at low or moderate risk). • Moderate CKD (GFR 30–59 ml/min/1.73 m^2^). • A calculated SCORE ≥ 5 and < 10%.
Moderate risk	• SCORE is ≥1 and < 5% at 10 years.
Low risk	• SCORE < 1%.

*ACS* acute coronary syndrome, *AMI* acute myocardial infarction, *BP* blood pressure, *CKD* chronic kidney disease, *CVD* cardiovascular disease, *DM* diabetes mellitus, *GFR* glomerular filtration rate, *PAD* peripheral artery disease, *SCORE* Systematic Coronary Risk Estimation, *TIA* transient ischaemic attack

**Table 2:** Target values for risk factors in patients with high and very high cardiovascular risk

Risk parameter	Target values for high-risk patients	Target values for very high-risk patients
BMI	< 27 kg/m^2^ (< 25 kg/m^2^)*	< 25 kg/m^2^
Waist circumference	men: < 102 cm, women: < 88 cm	men: < 94 cm, women: < 80 cm
Blood pressure	< 140/90 mmHg (diabetes: < 140/85 mmHg, nephropathy + proteinuria: < 130/80 mmHg)	< 140/90 mmHg (diabetes: < 140/85 mmHg, nephropathy + proteinuria: < 130/80 mmHg)
Total cholesterol	< 4.5 mmol/l	< 3.5 mmol/l
LDL-C	< 2.5 mmol/l (< 2.6 mmol/l)*	< 1.8 mmol/l
HDL-C	men: > 1.0 mmol/L, women: > 1.3 mmol/l (> 1.2 mmol/l)*	men: > 1.0 mmol/l, women: > 1.3 mmol/l (> 1.2 mmol/l)*
Triglycerides	< 1.7 mmol/l	< 1.7 mmol/l
HbA1c	< 7%	< 7%

* target value according to the 2016 European Guidelines on cardiovascular disease prevention in clinical practice. *BMI* body mass index, *HbA1c* glycated haemoglobin, *HDL-C* high-density lipoprotein cholesterol, *LDL-C* low density lipoprotein cholesterol

We conducted a cross-sectional study investigating the distribution of cardiovascular risk levels as defined in the study objectives and determined the proportion of patients who achieved the target level stratified by sex and risk level. Our data are presented primarily as raw case numbers and proportions. Data were analysed for categorical variables using chi-square tests.

Our study started in January 2019, and the data for this paper were collected until the end of 2020.

## Results

### Age and sex

Our study included 37,778 patients aged 40–65 years, with 14,944 (39.6%) men and 22,834 (60.4%) women. The mean age was 53.4 ± 6.95 years for men and 53.5 ± 7.03 years for women, with no significant difference between the sexes regarding mean age. The characteristics of the study population according to sex and age are presented in Table 3.

**Table 3:** Characteristics of the study population according to sex and age

Study population	Male	Female	Total
Number of involved patients	14,944	22,834	37,778
Proportion by sex	39.6%	60.4%	100.0%
Mean age (years)	53.4	53.5	53.4
Standard deviation of mean age (years)	6.95	7.03	7.00

### Distribution of cardiovascular risk levels

Of the study population, 37,298 patients had all the data needed to determine their cardiovascular risk category. Of these patients, 27.0% had low cardiovascular risk according to the 2016 European Guidelines on cardiovascular disease prevention in clinical practice [1] (men:14.5%, women: 35.2%) (Table 4). Of the participants, 18.5% were at medium risk (men: 18.9%, women: 18.2%). High cardiovascular risk was noted for 23.1% of patients (men: 27.1%, women: 20.5%). Very high-risk level was noted in 31.4% of participants (men: 39.6%, women: 26.1%). The difference in risk level distribution between the two sexes was significant (*p* < 0.001). The distribution of cardiovascular risk level by sex is presented in Table 4.

**Table 4:** Distribution of cardiovascular risk categories according to sex

Risk category	Male		Female		Total	
	N	%	N	%	N	%
Very high	5843	39.6%	5885	26.1%	11,728	31.4%
High	3995	27.1%	4615	20.5%	8610	23.1%
Medium	2788	18.9%	4100	18.2%	6888	18.5%
Low	2144	14.5%	7928	35.2%	10,072	27.0%
Total:	14,770	100.0%	22,528	100.0%	37,298	100.0%

### Target level achievement in the high cardiovascular-risk group

The therapeutic target values for each risk factor for patients at high cardiovascular risk are presented in Table 5.

**Table 5:** Success in achieving target values in our study and in the EUROASPIRE IV study

*High-risk patients*	Three Generations for Health Study			EUROASPIRE IV
	On target			
Risk factor and target value	Total	Male	Female	Total
Total cholesterol: < 4.5 mmol/l	**16.1%** (*n* = 1388/8610)	18.6% (*n* = 744/3995)	14.0% (*n* = 644/4615)	
LDL-cholesterol: < 2.5 mmol/l	**16.8%** (*n* = 1426/8478)	18.1% (*n* = 707/3916)	15.8% (*n* = 719/4562)	18.4% (*n* = 763/4137)
HDL-cholesterol: male: > 1.0 mmol/l, female: > 1.3 mmol/l	**82.2%** (*n* = 7075/8610)	90.3% (*n* = 3607/3995)	75.1% (*n* = 3468/4615)	
Triglyceride: < 1.7 mmol/l	**66.3%** (*n* = 5710/8610)	61.5% (*n* = 2455/3995)	70.5% (*n* = 3255/4615)	
Blood pressure: < 140/90 mmHg (diabetes: < 140/85 mmHg. Nephropathy + proteinuria: < 130/80 mmHg)	**63.4%** (*n* = 5439/8584)	56.8% (*n* = 2261/3984)	69.1% (*n* = 3178/4600)	44.7% (*n* = 2031/4540)
HbA1c < 7% (patients with type 2 diabetes)	**57.3%** (*n* = 110/192)	51.6% (*n* = 50/97)	63.2% (*n* = 60/95)	58.5% (*n* = 689/1177)
BMI < 27 kg/m^2^	**48.5%** (*n* = 4155/8570)	41.8% (*n* = 1665/3980)	54.3% (*n* = 2490/4590)	
BMI < 25 kg/m^2^	**31.1%** (*n* = 2667/8570)	22.6% (*n* = 899/3980)	38.5% (*n* = 1768/4590)	
Waist circumference: (male: < 102 cm, female: < 88 cm)	**44.4%** (*n* = 3378/7606)	55.1% (*n* = 1957/3549)	35.0% (*n* = 1421/4057)	36.1% (*n* = 1585/4392)

*n* number of cases reaching target value/total cases in the category

The target total cholesterol level under 4.5 mmol/l was achieved in 16.1% of high-risk patients (men: 18.6%, women: 14%) (Table 5). The target LDL-cholesterol level of less than 2.5 mmol/l was achieved in 16.8% of patients (men: 18.1%, women: 15.8%) in this group. The target HDL-cholesterol levels (men: > 1.0 mmol/l, women: > 1.3 mmol/l) were achieved in 82.2% of the total high-risk population (men: 90.3%, women: 75.1%). The target triglyceride level below 1.7 mmol/l was achieved in 66.3% of patients (men: 61.5%, women: 70.5%).

The BMI target of less than 27 kg/m^2^ was achieved by 48.5% of patients (men: 41.8%, women: 54.3%). Of this population, 31.1% (men: 22.6%, women: 38.5%) achieved a BMI below 25 kg/m^2^. Regarding waist circumference, 44.4% of high-risk patients (men: 55.1%, women: 35%) achieved the target (< 102 cm for men, < 88 cm for women).

The target blood pressure (< 140/90 mmHg, < 140/85 mmHg in diabetes mellitus, and < 130/80 mmHg in nephropathy + proteinuria) was achieved in 63.4% of patients in the high-risk group (men: 56.8%, women: 69.1%). Among high-risk patients with type 2 diabetes, 57.3% achieved HbA1c below 7% (men: 51.6%, women: 63.2%).

In the high-risk group, the differences between the two sexes regarding the rates of target achievement were significant for all but one parameter (*p* < 0.001). The difference in HbA1c target achievement between sexes was not significant due to the low number of cases (*p* = 0.104).

### Target level achievement in the very high cardiovascular-risk group

The therapeutic target values for patients at very high cardiovascular risk are presented in Table 6.

**Table 6:** Success in achieving target values in our own and in EUROASPIRE V

*Very high-risk patients*	Three Generations for Health Study			EUROASPIRE V
	On target			
Risk factor and target value	Total	Male	Female	Total (*n* = 8261)
Total cholesterol: < 3.5 mmol/l	**4.7%** (*n* = 550/11728)	6.1% (*n* = 355/5843)	3.3% (*n* = 195/5885)	
LDL-cholesterol: < 1.8 mmol/l	**8.0%** (*n* = 916/11463)	9.5% (*n* = 539/5687)	6.5% (*n* = 377/5776)	29%
HDL-cholesterol: male: > 1.0 mmol/l, female: > 1.3 mmol/l	**75.4%** (*n* = 8842/11728)	85.0% (*n* = 4964/5843)	65.9% (*n* = 3878/5885)	
Triglyceride: < 1.7 mmol/l	**55.0%** (*n* = 6450/11728)	51.8% (*n* = 3025/5843)	58.2% (*n* = 3425/5885)	
Blood pressure: < 140/90 mmHg (diabetes: < 140/85 mmHg, nephropathy + proteinuria: < 130/80 mmHg)	**49.9%** (*n* = 5842/11709)	42.8% (*n* = 2495/5834)	57.0% (*n* = 3347/5875)	58%
HbA1c < 7% (patients with type 2 diabetes)	**53.0%** (*n* = 2217/4183)	50.3% (*n* = 1066/2121)	55.8% (*n* = 1151/2062)	54% (*n* = 29% of all patients)
BMI < 25 kg/m^2^	**17.4%** (*n* = 2038/11698)	15.0% (*n* = 876/5829)	19.8% (*n* = 1162/5869)	18%
Waist circumference (male: < 102 cm, female: < 88 cm)	**29.4%** (*n* = 3166/10784)	39.3% (*n* = 2104/5348)	19.6% (*n* = 1062/5436)	41%
Waist circumference (male: < 94 cm, female: < 80 cm)	**14.3%** (*n* = 1543/10784)	21.4% (*n* = 1143/5348)	7.4% (*n* = 400/5436)	

*n* number of cases reaching target value/total cases in the category

Total cholesterol below 3.5 mmol/l was achieved in 4.7% of patients at very high risk (men: 6.1%, women: 3.3%) (Table 6). The target LDL-cholesterol level of less than 1.8 mmol/l was achieved in 8.0% of patients in this group (men: 9.5%, women: 6.5%). Target HDL-cholesterol (men: > 1.0 mmol/l, women: > 1.3 mmol/l) achievement was successful in 75.4% of the very high-risk population (men: 85.0%, women: 65.9%). The target triglyceride level below 1.7 mmol/l was achieved by 55% of patients (men: 51.8%, women: 58.2%). BMI below 25 kg/m^2^ was achieved by 17.4% of patients (men: 15.0%, women: 19.8%).

Regarding waist circumference, 14.3% of very high-risk patients (21.4% of men and 7.4% of women) achieved the target (< 94 cm for men and < 80 cm for women). In this group, a further 15.1% of patients (men: 17.9%, women: 12.2%) had a waist circumference between the target value and the more permissive target waist circumference (men: 94–102 cm, women: 80–88 cm).

The blood pressure target (< 140/90 mmHg, < 140/85 mmHg in diabetes mellitus, and < 130/80 mmHg in nephropathy + proteinuria) was reached by 49.9% of patients in the very high-risk group (men: 42.8%, women: 57.0%). Among very high-risk patients with type 2 diabetes, 53.0% achieved a HbA1c below 7% (men: 50.3%, women: 55.8%).

In the very high-risk group, the differences between the two sexes regarding the rates of target achievement were significant for all parameters (*p* < 0.001).

## Discussion

### LDL cholesterol

Achievement of the LDL cholesterol target among high-risk patients was 16.8%; approximately 5 out of 6 patients failed to reach an LDL cholesterol level below 2.5 mmol/l. This result is comparable with the results of the European Action on Secondary and Primary Prevention by Intervention to Reduce Events (EUROASPIRE) IV trial [4]. This trial investigated the success of achieving target levels in patients at high cardiovascular risk who were free of atherosclerotic cardiovascular disease in primary care in 14 European countries and involved more than 4000 patients. The results, published in 2016, revealed that a total of 18.4% of high-risk patients achieved the LDL-C target of less than 2.5 mmol/l, including both treated (with lipid-lowering medication) and untreated participants. Therefore, our study results are in line with the European results from 2016, albeit slightly less optimal.

In our study, 8.0% of very high-risk patients were successful in reaching the LDL-C target of 1.8 mmol/l. This suggests that, among our highest-risk patients, approximately 1 in 12 patients had adequate lipid target management. We compared these results with data from the 2019 EUROASPIRE V study involving more than 8000 patients in 27 European countries [7]. In this study, the achievement of target values in very high-risk patients undergoing a coronary event or coronary intervention was assessed between 6 and 24 months after the event/intervention. Among the patients, 29% were successful in achieving the LDL-C target of 1.8 mmol/l. In this context, our data suggest a significant delay in success of very high-risk patients compared with other European countries, even if the population included in our study was not linked to a specific coronary event/intervention and a period close to it.

Furthermore, we observed that the success rate of achieving the target LDL-C value was significantly higher among men than women in both risk groups in our study.

There is no doubt that LDL-C target achievement in both high-risk and very high-risk patients is markedly inadequate in Europe, especially in Hungary.

### HDL cholesterol and triglyceride

HDL-C and triglyceride levels are also determinants of cardiovascular risk, although their importance as targets for intervention is inferior to LDL-C [1]. Nevertheless, their evolution is worth investigating, as these parameters are part of the overall picture of the effectiveness of dyslipidaemia therapy. In our study, HDL-C target attainment was > 80% in the high-risk group and > 75% in the very high-risk group, with significantly better treatment efficacy in men than in women. Compared with the results of a 2016 Hungarian study, HDL-C target attainment improved in both risk groups over the past 4–5 years from 66.7 to 82.2% in high-risk patients and from 68 to 75.4% in very high-risk patients [5].

The picture is less favourable for triglyceride targets, with just over 66% of high-risk individuals and 55% of very high-risk individuals achieving triglyceride levels below 1.7 mmol/l. In the 2016 Hungarian study, these rates were 47.8 and 45%, respectively; therefore, there was an improvement in the achievement of target values for this parameter in Hungary [5]. Regarding triglyceride target achievement, the treatment outcomes of women were significantly more favourable compared with men in our study.

### Blood pressure

In our study, the blood pressure target was achieved in 63.4% of high-risk patients and in just under 50% of very high-risk patients. In both risk groups, women had significantly better outcomes of care. Compared with Europe overall, blood pressure target attainment in our study among high-risk individuals showed a more favourable picture than the 2016 results of EUROASPIRE IV, which reflected the European average (63.4% vs 44.7%). Moreover, among very high-risk patients, target attainment in our study was lower than the European data published in EUROASPIRE V in 2019 (49.9% vs 58%, respectively) [4, 7].

### BMI

Achievement of the target BMI (< 27 kg/m^2^) exceeded 48% in the high-risk group; however, in the very high-risk group, where the target was below 25 kg/m^2^, the success rate was only 17.4%. This rate is in line with data from EUROASPIRE V 2019, where 18% of very high-risk patients had a BMI below 25 kg/m^2^ [7].

### Abdominal circumference

Regarding abdominal circumference values among high-risk patients, almost 45% of women had a circumference < 88 cm and men < 102 cm, while the 2016 data from EUROASPIRE IV indicated that this proportion was only 36.1% for the average of the European countries included in that study [4].

When evaluating the results for the very high-risk group, just over 14% of patients achieved the target abdominal circumference (women < 80 cm, men < 94 cm), and the success rate for women was extremely low at only 7.4%. Regarding the success rate of reaching the 88 cm and 102 cm abdominal circumference limits for women and men, respectively, the success rates were 39.3 and 19.6% for men and women, respectively, for a total success rate of 29.4%.

In the EUROASPIRE V study population, which is considered identical in terms of very high risk, the proportion of patients with an abdominal circumference below the central obesity value was 41%, which is significantly higher than our national data [7]. Analysis of the data indicates that the achievement of BMI and abdominal circumference targets, especially in the very high-risk population, is highly unfavourable according to the European study results; however, the Hungarian data show an even more severe picture of the prevalence and management of obesity.

Among high-risk patients, 57.3% of patients with type 2 diabetes had a HbA1c level below 7%, which is close to the European average of 58.5% in EUROASPIRE IV [4]. Among very high-risk type 2 diabetic patients in our study, the proportion of patients with HbA1c below 7% was 53%, which is almost identical to the 54% reported in EUROASPIRE V [7].

### Meaning of our results, clinical relevance

Our results suggest that Hungary is significantly below the European average in two areas in terms of achieving target values for the most vulnerable patients at very high cardiovascular risk, namely abdominal circumference and LDL-C.

The success rate for achieving the target abdominal circumference (male: < 102 cm, female: < 88 cm) in our study was 29.4%, compared with the European average of 41%; the success rate for women in Hungary was especially unfavourable at 19.6%. This highlights the inadequacy of the management of obese and overweight patients in Hungary, which exceeds the European extent of this problem. Given the role of abdominal obesity in the development of many additional cardiovascular risk factors (e.g., dyslipidaemia, type 2 diabetes mellitus, hypertension) [10, 11], appropriate management and improvement of the success rate of achieving the target abdominal circumference is essential for reducing cardiovascular risk at the population level.

Non-pharmacological treatment and lifestyle modifications play a crucial role in the management of abdominal obesity, which requires complex support for the patient (e.g., nutrition therapy, exercise therapy, stress management), for which a multidisciplinary approach and a broader spectrum of professionals need to be easily accessible to primary care [12, 13]. Our results indicate that these conditions are not currently met in the Hungarian health care system.

The very low success rate of achieving the LDL-C target level (< 1.8 mmol/l) in very high-risk patients in Hungary today, as indicated by our study (8% of patients at target compared with 29% in Europe), highlights a major system-level problem in Hungary. If we consider the two previous Hungarian studies (completed in 2011 and 2016) involving smaller populations [7], the success rate of reaching the LDL-C target of 1.8 mmol/L was slightly better (10.7 and 15.8%) than the 8% success rate reported in our study, indicating a break in the slightly improving trend in Hungary among very high-risk patients.

The high prevalence of abdominal obesity may also play a role in the unfavourable development of LDL-C values; however, for this parameter, the ineffectiveness of lipid-lowering drug therapy is also likely [10]. There is also the question of low dosing of statin monotherapy, insufficient frequency of combined cholesterol-lowering therapy, and low levels of patients’ drug adherence, as suggested by several Hungarian and international studies [14–17].

Achievement of the blood pressure target value in the very high-risk group was also less successful in our study than in the European study (49.9% vs. 58%, respectively); however, the difference was not as striking as in the abdominal circumference and LDL-C targets. At the same time, the lower achievement rate of the target blood pressure in Hungary reflects the high prevalence of abdominal obesity as a major risk factor for hypertension in the very high-risk population, which further highlights the need to improve obesity management [10].

### Future perspectives

Based on the results of our study, we identified the management of abdominal obesity and the reduction of LDL-C levels with lifestyle modifications and drug therapy in patients at very high cardiovascular risk as priority areas for the development of cardiovascular prevention in Hungary. To improve the success of achieving these goals, general practice partnerships should play a key role in these areas, where multidisciplinary teams (e.g., exercise therapists, nutrition therapists, health psychologists) can be established to support lifestyle changes.

Furthermore, it is necessary to increase the effectiveness of lipid-lowering therapy, both through lifestyle changes and via full implementation of modern therapeutic guidelines and techniques to increase patient adherence in both primary and specialist care [18]. From a financial point of view, patients should have easy access to combination statin-ezetimibe therapy and even PCSK-9 inhibitors in Hungary to ensure that improvements in target attainment can be achieved, especially given the further reduction in the LDL-C target values since the publication of the 2016 Joint European Guideline [19].

### Study limitations

A limitation of our study is that patients were recruited using a consecutive method in general practices rather than a randomised approach. The consecutive method is more compatible with the day-to-day routine operations of general practices, and the large study population obtained confirms this, which partly compensates for the lack of randomised selection.

Furthermore, our results may be influenced by the fact that the success of achieving the target was examined in a population of GP practices where GPs themselves may be more motivated and committed to cardiovascular prevention, as enrolment in the study was voluntary on the part of GPs and required additional work over and above the normal daily activities of the practice.

Our study was a descriptive, cross-sectional study, which provided less opportunity to establish cause and effect relationships. However, we believe that describing the cardiovascular risk levels and target attainment rates of such a large population provides very important data on the preventive work of the health care system and primary care and contributes significantly to identifying gaps and target areas for improvement.

## Conclusions

In summary, our survey data indicated that, in Hungary, LDL-C target achievement was significantly lower than the European average, which was also low. Blood pressure target attainment in high-risk patients was slightly higher than the European average and slightly lower the average in very high-risk patients. Patients with type 2 diabetes achieved HbA1c levels below 7% at a rate that was in line with the average European success rate.

A waist circumference of less than 88 cm for women and 102 cm for men was achieved by just under one-third of very high-risk patients in our study, which is below the European average reported in EUROASPIRE V [4].

These data clearly underline the importance of central obesity in the development of cardiovascular risk in addition to the very low effectiveness of obesity management in both European and Hungarian contexts. To improve the success rates of target achievement in both abdominal obesity and LDL-C reduction, priority should be given to multidisciplinary teams (e.g., exercise therapists, nutrition therapists, health psychologists) that can provide appropriate support for lifestyle changes in these patients. These teams would be optimally located in general practice partnership settings. Furthermore, there is a need to enhance the effectiveness of lipid-lowering drug therapy through more stringent implementation of relevant guidelines in practice and increased patient adherence.

## Data Availability

The data sets used and/or analysed in the current study are available from the corresponding author upon reasonable request.

## Abbreviations

ACS: acute coronary syndrome
AMI: acute myocardial infarction
BMI: body mass index
BP: blood pressure
CKD: chronic kidney disease
CVD: c*ardiovascular disease*
DM: diabetes mellitus
EUROASPIRE: European Action on Secondary and Primary Prevention by Intervention to Reduce Events
GFR: glomerular filtration rate
GOKVI: Gottsegen György National Cardiovascular Institute
GPs: General practitioners
HCCC: Hungarian Cardiovascular Consensus Conference
HDL-C: high-density lipoprotein cholesterol
HbA1c: glycated haemoglobin
LDL-C: low-density lipoprotein cholesterol
PAD: peripheral artery disease
SCORE: Systematic Coronary Risk Estimation
TIA: transient ischaemic attack
